# Supplementary material for: Uterine Flushing Fluid-Derived Let-7b Targets CXCL10 to Regulate Uterine Receptivity in Goats during Embryo Implantation
Source: Int J Mol Sci. 2023 Feb 1;24(3):2799. doi: 10.3390/ijms24032799 (PMC9917504; doi:10.3390/ijms24032799)
Supplement: Supplementary file 1 [file ijms-24-02799-s001.zip › ijms-2079180-supplementary.pdf]

Table S1. Differentially expressed miRNA.

Gene ID	log2 (D18P / D15P)	Qvalue (D18P / D15P)	log2 (D15P / D5P)	Qvalue (D15P / D5P)	log2 (D18P / D5P)	Qvalue (D18P / D5P)
chi-let-7a-5p			-4.316507819	7.91E-05	-9.638435914	1.23E-05
chi-let-7b-5p	-11.32811389	6.14E-18	2.550036765	1.47E-29	-8.77807713	0.00213525
chi-let-7c-5p	-8.566054038	5.70E-04	8.566054038	1.74E-06		
chi-let-7f-5p	8.761551232	1.06E-05			8.761551232	1.32E-09
chi-let-7i-5p	8.682994584	1.85E-05			2.343144581	1.37E-08
chi-miR-106b-3p			-10.30833903	1.82E-08	-10.30833903	1.17E-08
chi-miR-127-3p	-8.335390355	0.006179092	8.335390355	9.85E-05		
chi-miR-1306-3p	-2.737665762	1.23E-04	2.200164615	2.34E-12	-0.537501147	0.047456169
chi-miR-136-5p			-10.52061868	1.27E-09	-10.52061868	8.26E-10
chi-miR-151-3p	4.972979786	1.55E-11	-7.105908509	1.42E-28	-2.132928723	0.027668333
chi-miR-16a-5p			-4.781359714	5.91E-07	-10.10328781	7.26E-08
chi-miR-191-5p			-9.364134655	1.08E-04	-9.364134655	8.70E-05
chi-miR-193b-3p	9.214319121	2.27E-07			4.459431619	5.78E-13
chi-miR-199a-3p	-8.921840937	5.70E-04	8.921840937	1.74E-06		
chi-miR-199a-5p	-2.730406106	0	-0.382439506	3.44E-235	-3.112845612	0
chi-miR-199b-5p	-1.994926697	4.09E-108	-2.105263596	0	-4.100190294	0
chi-miR-199c-5p	12.31967212	7.52E-44			12.31967212	3.80E-68
chi-miR-204-5p	-9.036173613	3.22E-04	9.036173613	6.67E-07		
chi-miR-21-5p	-2.395301281	0.314781462	-4.77853877	7.50E-19	-7.173840051	1.32E-21
chi-miR-218			-8.885696373	0.001176072	-8.885696373	9.99E-04
chi-miR-25-3p	8.422064766	1.04E-04	-7.761551232	0.076004804	0.660513534	4.66E-05
chi-miR-26a-	1.33560303	6.26E-08	0.6567952	0.006721222	1.9923982	2.04E-23

5p	2		45		77	
chi-miR-26b-5p	9.269126679	1.36E-07			9.269126679	8.24E-13
chi-miR-30c-5p			-9.296916207	1.57E-04	-9.296916207	1.29E-04
chi-miR-320-3p	-9.796039609	1.96E-06	9.796039609	1.50E-10		
chi-miR-331-3p	-9.859534786	1.15E-06	-1.604500366	0.014935635	-11.46403515	4.43E-18
chi-miR-3431-5p			-10.29806257	2.51E-08	-10.29806257	1.60E-08
chi-miR-345-3p			-9.786269628	5.18E-06	-9.786269628	3.72E-06
chi-miR-451-5p			-9.098032083	3.50E-04	-9.098032083	2.89E-04
chi-miR-7-5p	8.515699838	5.88E-05			8.515699838	2.14E-08
chi-miR-92a-3p	9.098032083	6.71E-07	-8.885696373	0.001176072	0.21233571	1.26E-05
novel-chi-miR1172-5p	12.64227738	1.72E-52	-8.098032083	0.024716259	4.544245296	7.44E-128
novel-chi-miR1207-5p	7.515699838	0.006301391			7.515699838	7.77E-05
novel-chi-miR1273-3p	1.76106127	0.006436468			8.330916878	1.51E-07
novel-chi-miR1344-5p	-1.246121238	1.00E-08	13.19521839	9.22E-85	11.94909716	5.33E-56
novel-chi-miR1412-5p	0.951737925	8.27E-17	2.707819249	1.94E-37	3.659557173	2.66E-110
novel-chi-miR1453-5p	11.87382857	3.48E-34			11.87382857	9.45E-54
novel-chi-miR1612-5p	8.21916852	3.29E-04			8.21916852	4.10E-07
novel-chi-miR1739-3p	6.146133512	2.08E-298	2.379213836	5.26E-06	8.525347347	0
novel-chi-miR1851-5p	-8.303780748	0.001915172	8.303780748	1.23E-05		
novel-chi-miR1878-5p	0.986315189	9.39E-100	4.409956588	7.11E-223	5.396271777	0
novel-chi-miR2002-5p	13.80413102	9.55E-99	-8.764871591	0.003803483	5.03925943	6.84E-279
novel-chi-miR2265-3p	4.479780264	2.37E-08			9.479780264	2.60E-14
novel-chi-miR434-3p	2.113228978	8.57E-24	-2.127111918	4.66E-05	-0.01388294	2.70E-24
novel-chi-miR494-5p	4.837627933	1.42E-10	-1.339850003	0.965187582	3.49777793	2.75E-19

novel-chi-miR893-3p	2.997603343	3.58E-66	1.471640676	3.53E-10	4.469244019	5.06E-156
novel-chi-miR920-3p	5.120142363	3.31E-35			11.68999797	7.68E-49

Table S2. Reads quality statistics after sample filtration (mRNA).

Sample	Total Raw Reads (million)	Total Clean Reads (million)	Total Clean Bases (Gb)	Clean Reads Q20 (%)	Clean Reads Q30 (%)	Clean Reads Ratio (%)
P_D5-1	114.86	111.93	11.19	98.45	95.39	97.45
P_D5-2	114.94	112.28	11.23	98.12	94.42	97.68
P_D15-1	114.94	112.82	11.28	98.72	96.04	98.15
P_D15-2	114.94	112.34	11.23	98.69	95.97	97.74
P_D18-1	114.94	111.99	11.2	98.46	95.36	97.43
P_D18-2	114.94	112.94	11.29	97.62	93.37	98.26

Table S3. Reads quality statistics after sample filtration (small RNA).

Sample	Sequence Type	Raw Tag Count	Low Quality Tag Count	Invalid Adapter Tag Count	Poly A Tag Count	Short Valid Length Tag	Clean Tag Count	Q20 of Clean Tag	Percentage of Clean Tag (%)
P_D5-1	SE50	3.77E+07	128949	469080	554	907085	3.62E+07	99	96.01
P_D5-2	SE50	3.77E+07	135380	559527	586	2527293	3.45E+07	98.9	91.46
P_D15-1	SE50	3.27E+07	90699	532297	1319	905071	3.12E+07	98.6	95.32
P_D15-2	SE50	3.17E+07	83857	514662	1745	1421128	2.96E+07	98.6	93.61
P_D18-1	SE50	3.77E+07	129863	507805	1786	1322729	3.58E+07	98.9	94.8
P_D18-2	SE50	4.23E+07	144057	1046304	5196	1.10E+07	3.00E+07	99	71.08

Table S4. Comparison information statistical table (mRNA).

Sample	Total Clean Reads (M)	Total Mapping(%)	Uniquely Mapping(%)
P_D5-1	111.93	72.88	46.63
P_D5-2	112.28	72.36	48.52
P_D15-1	112.82	70.03	48.85
P_D15-2	112.34	40	25.22
P_D18-1	111.99	52.01	32.18

P_D18-2	112.94	16.16	9.6

Table S5. Comparison information statistical table (small RNA).

Sample	Total Clean Reads (M)	Total Mapping(%)
P_D5-1	34.56	96.31
P_D5-2	32.93	97.86
P_D15-1	29.74	94.7
P_D15-2	28.26	94.72
P_D18-1	34.13	88.89
P_D18-2	28.64	82.62
